# Coenzyme Q_10_ supplementation improves the motor function of middle-aged mice by restoring the neuronal activity of the motor cortex

**DOI:** 10.1038/s41598-023-31510-1

**Published:** 2023-03-15

**Authors:** Ritsuko Inoue, Masami Miura, Shuichi Yanai, Hiroshi Nishimune

**Affiliations:** 1 Laboratory of Neurobiology of Aging, Tokyo Metropolitan Institute for Geriatrics and Gerontology, 35-2 Sakaecho, Itabashi-Ku, Tokyo 173-0015 Japan; 2 Laboratory of Memory Neuroscience, Tokyo Metropolitan Institute for Geriatrics and Gerontology, 35-2 Sakaecho, Itabashi-Ku, Tokyo 173-0015 Japan; 3 Saitama Central Hospital, 2177 Kamitome, Miyoshicho, Iruma-Gun, Saitama 354-0045 Japan; 4 Department of Applied Biological Science, Tokyo University of Agriculture and Technology, 3-8-1 Harumicho, Fuchu-Shi, Tokyo 183-8538 Japan

**Keywords:** Neural ageing, Long-term potentiation, Motor cortex

## Abstract

Physiological aging causes motor function decline and anatomical and biochemical changes in the motor cortex. We confirmed that middle-aged mice at 15–18 months old show motor function decline, which can be restored to the young adult level by supplementing with mitochondrial electron transporter coenzyme Q_10_ (CoQ_10_) as a water-soluble nanoformula by drinking water for 1 week. CoQ_10_ supplementation concurrently improved brain mitochondrial respiration but not muscle strength. Notably, we identified an age-related decline in field excitatory postsynaptic potential (fEPSP) amplitude in the pathway from layers II/III to V of the primary motor area of middle-aged mice, which was restored to the young adult level by supplementing with CoQ_10_ for 1 week but not by administering CoQ_10_ acutely to brain slices. Interestingly, CoQ_10_ with high-frequency stimulation induced NMDA receptor-dependent long-term potentiation (LTP) in layer V of the primary motor cortex of middle-aged mice. Importantly, the fEPSP amplitude showed a larger input‒output relationship after CoQ_10_-dependent LTP expression. These data suggest that CoQ_10_ restores the motor function of middle-aged mice by improving brain mitochondrial function and the basal fEPSP level of the motor cortex, potentially by enhancing synaptic plasticity efficacy. Thus, CoQ_10_ supplementation may ameliorate the age-related decline in motor function in humans.

## Introduction

The age-related decrease in motor function can be caused by a loss of muscle mass and strength (sarcopenia), denervation of neuromuscular junctions (NMJs), loss of motor neurons in the spinal cord, and reduced function of the brain motor cortex. In mice, a decline in motor function was observed in middle-aged mice (15 months old); earlier than the drop in survival rate that typically occurs at approximately 24 months of age^1–3^. In the human motor cortex, physiological aging causes cortical atrophy, altered excitability, and decreased neurotransmitter levels ^4–6^. Voluntary activation of skeletal muscles is impaired during aging, especially in elderly individuals who are weak or in poor physical condition^5^. Elderly individuals show a decrease in the firing rate of lower motor neurons, which may be related, at least in part, to decreased activity of the motor cortex. In rodents, middle-aged mice show motor function impairment and increased phosphorylation of α-synuclein and a decreased level of vesicular glutamate transporter 1 (VGluT1) in motor cortex compared to those of young adult mice^1,7^. These impairments in the motor cortex may be part of the underlying mechanism that leads to age-related motor function decline.

Brain aging also causes deficits in the mitochondrial oxidative phosphorylation system, producing ATP necessary to fulfill brain neuronal functions^8^. The basal ganglia putamen of old rhesus monkeys showed decreased mitochondrial functions of ATP synthesis and calcium buffering, which correlated with age-related motor deficits^8^. Interestingly, mitochondrial function measured by peroxide production was higher in synaptic mitochondria than in nonsynaptic mitochondria in rat brains. Furthermore, mitochondrial respiration function decreased significantly with age only in synaptic mitochondria but not in nonsynaptic mitochondria among 14- and 17-month-old mice compared to those of 3-month-old mice^9,10^. These data suggest an age-related functional decline of mitochondria located at synapses in the brain. Mechanistically, mitochondrial oxidative phosphorylation requires the electron transporter coenzyme Q. In mice, coenzyme Q_9_ and Q_10_ are used to transport electrons from complexes I and II to complex III for ATP synthesis^11–13^. The level of coenzyme Q_10_ (CoQ_10_) decreases during aging in rodents and humans^1,14–16^. Interestingly, exogenous administration of CoQ_10_ ameliorated motor impairment and the brain mitochondrial respiration rate in aged mice^1,17^.

These studies demonstrate the age-related decline in motor functions, motor cortex functions, and synaptic mitochondrial functions. However, further research is needed to reveal what kind of electrophysiological impairments underlie the age-related decline in motor functions and to develop intervention methods to rescue the motor function and motor cortex neuronal activity of elderly or aged animals. This study tested whether the age-related decline in motor functions could be reversed by supplementing the mitochondrial component that decreases during aging. CoQ_10_ was supplemented by drinking water in middle-aged mice, which led to motor function improvements. To investigate the mechanism activated by CoQ_10_, we measured muscle strength, brain mitochondrial function, and electrophysiological activity in the motor cortex of middle-aged mice supplemented with CoQ_10_.

## Results

### CoQ_10_ supplementation reversed age-related decline in motor function

**Figure 1:**
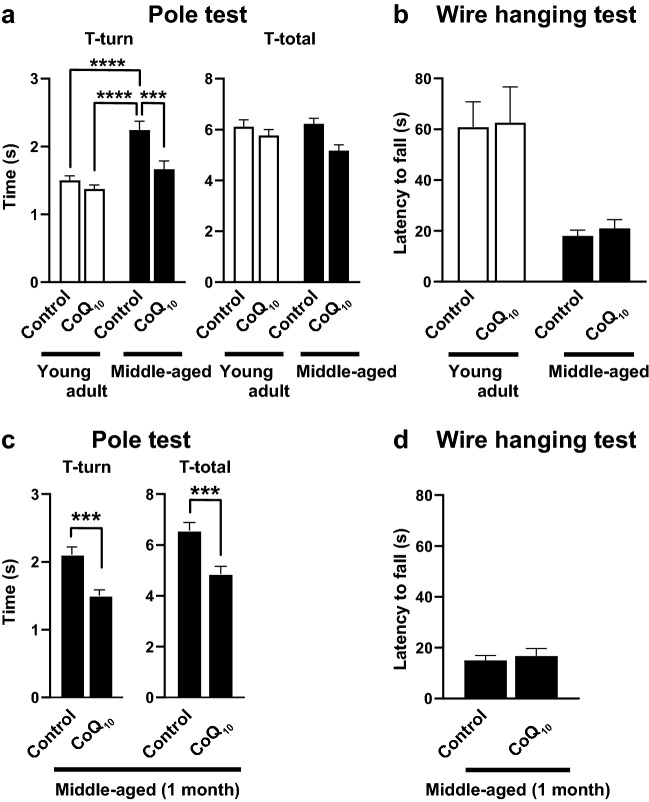
CoQ_10_ supplementation by drinking water restored motor function in middle-aged mice. Motor function was evaluated by the time to complete each aspect of the pole test. A T-turn represents the time required for a mouse to orient the body and feet downward at the top of a vertical pole. T-total represents the time required for the mouse to complete the T-turn and climb down to the experimental cage floor. (**a**) Pole test latency of young adult and middle-aged mice treated with drinking water supplemented with or without CoQ_10_ for 10–13 days. Young adult mice (6 months old; control, *n* = 20; CoQ_10_, *n* = 20) and middle-aged mice (15 months old; control, *n* = 20; CoQ_10_,* n* = 20) were used in the test (****p < 0.0001; ***p < 0.001, two-way ANOVA with Bonferroni's multiple comparison test). (**b**) Wire hanging latency of young adult and middle-aged mice supplemented with CoQ_10_ for 1 week compared to age-matched controls. The latency to fall did not show a difference with CoQ_10_ supplementation but was shortened by aging (*n* = 20 in each group of young adult control and CoQ_10_ and middle-aged control and CoQ_10_; no significant interaction, two-way ANOVA). (**c**) Middle-aged mice supplemented with CoQ_10_ for 33–36 days (approximately 1 month) showed improvement of pole test latency compared to age-matched controls (16-month-old; control, *n* = 20, CoQ_10_, *n* = 19; ***p < 0.001, Welch's *t* test). (**d**) Wire hanging latency of middle-aged mice supplemented with CoQ_10_ for 1 month compared to age-matched controls (16-month-old; control, *n* = 20; CoQ_10_,* n* = 19; no significant difference, Welch's *t* test). Values are expressed as the mean ± standard error of the mean (SEM) of independent experimental groups. For details of the data, see Supplementary Table 3.

We compared the motor function of young adult mice (6 months old) and middle-aged mice (15–16 months old) by performing the pole test following supplementation with water-soluble nanoformula-type CoQ_10_ by drinking water (150 μM, 40SP, Petroeuroasia) for 10–13 days or without supplementation (Fig. 1a). The pole test is used to evaluate motor coordination deficit^18–20^ by measuring the time required for mice to orient their body and feet completely downward at the top of a vertical pole (T-turn) and the total time to descend to the floor of the experimental cage (T-total). The 15-month-old middle-aged mice required a significantly longer time for the T-turn than the young adult mice (young adult control, 1.50 ± 0.07 s, middle-aged control, 2.24 ± 0.13 s). CoQ_10_ supplementation in middle-aged mice improved motor function (T-turn) by 25.76% to a level similar to that of the young adult controls (Fig. 1a left). There was a significant interaction between CoQ_10_ supplementation and age, a significant main effect of supplementation, and a significant main effect of age (Fig. 1a left; the interaction between supplementation and age *p* = 0.0296, *F* (1,76) = 4.919; the main effect of supplementation *p* = 0.0009, *F* (1, 76) = 11.95; the main effect of age *p* < 0.0001, *F* (1, 76) = 25.77, two-way ANOVA; young adult control compared to middle-aged control *p* < 0.0001; young adult CoQ_10_ compared to middle-aged control, *p* < 0.0001; middle-aged control compared to middle-aged CoQ_10_, *p* = 0.0008, Bonferroni’s multiple comparison test). Measurements of the total time (T-total) revealed a significant main effect of supplementation but did not show any main effect of age or a supplementation-age interaction (Fig. 1a right; the interaction between supplementation and age *p* = 0.1386, *F* (1,76) = 2.240; the main effect of supplementation *p* = 0.0043, *F* (1, 76) = 8.686, two-way ANOVA). The age-dependent decline in motor function in the pole test and recovery by water-soluble nanoformula-type CoQ_10_ supplementation is consistent with a previous study^1^.

In this study, we also tested the effect of CoQ_10_ supplementation in young adult mice to reveal whether the beneficial effect of CoQ_10_ is an age-specific effect or a general effect. Interestingly, CoQ_10_ supplementation did not change the T-turn of young adult mice (Fig. 1a left; young adult control compared to young adult CoQ_10_, *p* > 0.9999, Bonferroni's multiple comparison test) and revealed the significant main effect of supplementation on the T-total without the main effect of age or the supplementation-age interaction (Fig. 1a right). These results revealed that the beneficial effect of CoQ_10_ supplementation is stronger in middle-aged mice showing a decline in motor function.

We analyzed whether CoQ_10_ supplementation for 1 week affects muscle strength by measuring the wire hanging latency of young adult and middle-aged mice with or without CoQ_10_ supplementation. There was no interaction between CoQ_10_ supplementation and age or main effect of supplementation. However, there was a significant main effect of age, suggesting an age-related decline in muscle strength (Fig. 1b; the interaction between supplementation and age *p* = 0.9440, *F* (1, 76) = 0.0050; the main effect of age *p* < 0.0001, *F* (1,76) = 22.42, two-way ANOVA). CoQ_10_ supplementation did not affect the wire hanging latency of either young adult or middle-aged mice.

Furthermore, we tested the effect of extended CoQ_10_ supplementation by drinking water for approximately 1 month in middle-aged mice. This longer-term treatment also improved the pole test latency (Fig. 1c; T-turn by 28.62%, *p* = 0.0002, *t* (34) = 4.253; T-total by 25.94%, *p* = 0.0004, *t* (36.93) = 3.875, Welch's *t* test), suggesting that the beneficial effect of CoQ_10_ supplementation is maintained and there is no desensitization during the first month. In addition, there was no significant difference between the wire hanging latency of middle-aged mice with or without CoQ_10_ supplementation for 1 month (Fig. 1d; *p* = 0.6387,* t* (31.63) = 0.4741, Welch's *t* test). These results suggest no correlation between muscle strength and the improved motor function caused by CoQ_10_ supplementation.

### CoQ_10_ supplementation improved brain mitochondrial respiration in middle-aged mice

**Figure 2:**
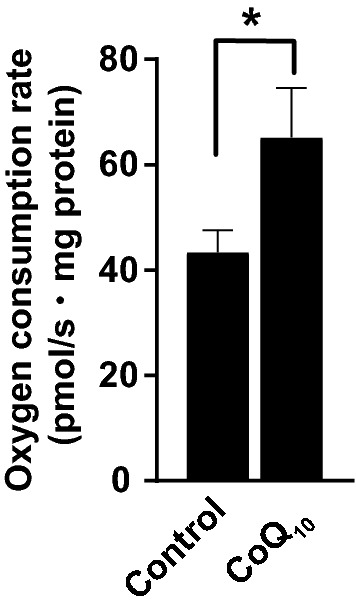
CoQ_10_ supplementation by drinking water concurrently restored brain mitochondrial respiration in middle-aged mice. The oxygen consumption rate (OCR) of the brain mitochondrial fraction was measured using high-resolution respirometry (Oxygraph-2k). Middle-aged mice were used in this measurement following CoQ_10_ supplementation for 40–76 days (control, *n* = 17; CoQ_10_, *n* = 17; *p < 0.05, Welch's *t* test). Values are expressed as the mean ± SEM of independent experimental groups. For details of the data, see Supplementary Table 3.

A previous study tested the effect of water-soluble nanoformula-type CoQ_10_ on motor and brain mitochondrial functions in different sets of animals and did not confirm them concurrently in the same animal^1^. In the current study, water-soluble nanoformula-type CoQ_10_ was administered to middle-aged mice for more than 1 month, and behavioral tests and measurements of brain mitochondrial function were performed in the same animals. A brain mitochondrial fraction was purified to measure the NADH-dependent (complex I-mediated) mitochondrial oxygen consumption rate (OCR). The brain mitochondrial OCR increased significantly in the CoQ_10_-supplemented middle-aged mice compared to that of the age-matched non-drug controls (Fig. 2; *p* = 0.0459,* t* (22.23) = 2.115, Welch's* t* test). These results suggested that brain mitochondrial function and motor function were concurrently restored in middle-aged mice supplemented with CoQ_10_ by drinking water.

### CoQ_10_ supplementation restored motor cortex neuronal activity

CoQ_10_ supplementation improved the motor function of middle-aged mice without enhancing muscle strength. Therefore, we investigated whether water-soluble nanoformula-type CoQ_10_ (Aqua Q_10_L10-NF) supplementation affects neuronal activity in the motor cortex. Synaptic mitochondria in the brain show age-related functional decline^9,10^. Brain mitochondrial activity is critical for maintaining normal synaptic function^21^.

**Figure 3:**
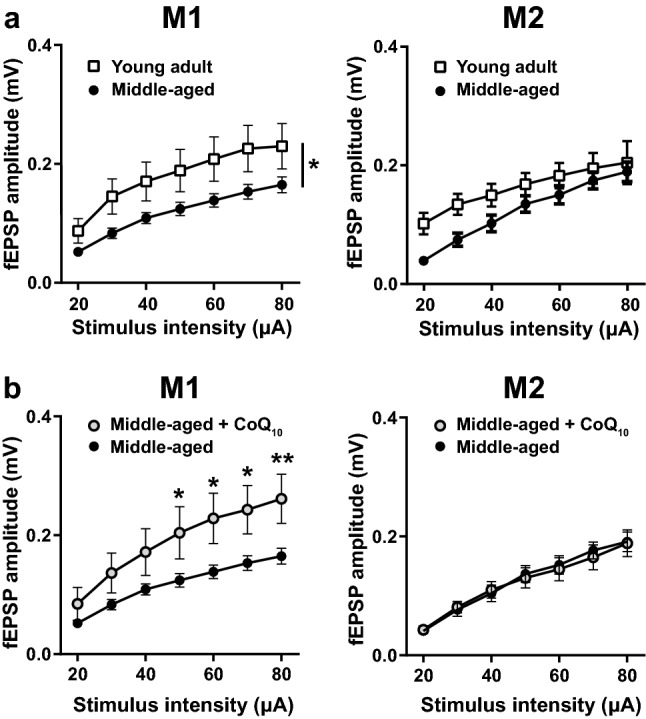
An age-related decline in synaptic transmission in the motor cortex of middle-aged mice and an improvement with CoQ_10_ supplementation by drinking water. The field excitatory postsynaptic potentials (fEPSPs) in layer V of the primary motor (M1) and secondary motor (M2) cortices were recorded separately using a multi-electrode array and stimulating the pathway from layers II/III to layer V with a single glass electrode. (**a**, left) fEPSP amplitudes in the layer V of M1 region showed a significant decrease with age (young adult, *n* = 14 slices from 5 mice; middle-aged, *n* = 29 slices from 10 mice; *p < 0.05). (**a**, right) In the M2 region, there were no significant differences between the young adult and middle-aged groups (young adult, *n* = 9 slices from 4 mice; middle-aged, *n* = 26 slices from 10 mice). (**b**, left) The fEPSP amplitudes in the layer V of M1 region of middle-aged mice supplemented with CoQ_10_ for 1 week were significantly higher than those of age-matched controls in the range of 50–80 μA current stimuli (middle-aged + CoQ_10_, *n* = 11 slices from 5 mice; middle-aged, *n* = 29 slices from 10 mice; **p < 0.01, *p < 0.05, Bonferroni's multiple comparison test). (**b**, right) There were no significant differences between fEPSP amplitudes of CoQ_10_-treated middle-aged mice and age-matched controls in the layer V of M2 region (middle-aged + CoQ_10,_ *n* = 16 slices from 5 mice; middle-aged,* n* = 26 slices from 10 mice). The middle-aged control data in (**b**) are identical to those in (**a**). Values are expressed as the mean ± SEM of independent experimental groups. Statistical analyses were performed using two-way repeated-measures ANOVA. For details of the data, see Supplementary Table 3.

The motor cortex is anatomically and functionally divided into the primary motor (M1) and the secondary motor (M2) cortices. In this study, we analyzed the two motor regions separately. We prepared cortical slices, stimulated layers II/III, and recorded the responses from layer V neurons to analyze the major intralaminar excitatory connection between layers II/III and V in the motor cortex^22–28^. fEPSP amplitudes were significantly reduced on average by 35.30 ± 1.85% in the M1 region of middle-aged mice compared to young adult mice in the tested range of 20–80 μA current stimuli (Fig. 3a left). The field excitatory postsynaptic potential (fEPSP) amplitude of young adult mice and middle-aged mice, both without CoQ_10_ supplementation, showed no interaction but a significant effect of age by two-way repeated-measures ANOVA (Fig. 3a left; the interaction between stimulus intensity and age *p* = 0.2778, *F* (6, 246) = 1.257; the main effect of age* p* = 0.0231,* F* (1, 41) = 5.572). These results suggested that physiological aging altered neuronal activity in the connection between layers II/III and V in the motor cortex. However, there was no significant interaction or main effect of age by two-way repeated-measures ANOVA in the fEPSP amplitude in the M2 region between young adult and middle-aged mice (Fig. 3a right; the interaction between stimulus intensity and age *p* = 0.0941, *F* (6, 198) = 1.835; the main effect of age* p* = 0.1107,* F* (1, 33) = 2.687, two-way repeated-measures ANOVA). These results suggest an age-dependent decline in neuronal activity in a motor cortex region-specific manner.

Next, we tested whether CoQ_10_ supplementation affects the age-related decline in fEPSP amplitude in middle-aged mice. Cortical slices were prepared from the brains of middle-aged mice supplemented with CoQ_10_ for 1 week and compared to those of age-matched controls without CoQ_10_ supplementation. fEPSP amplitudes were significantly increased on average by 61.51 ± 1.12% in the M1 region of CoQ_10_-supplemented middle-aged mice compared to middle-aged control mice in the range of 50–80 μA current stimuli (Fig. 3a left; Middle-aged compared to Middle-aged CoQ_10_, 50–80 μA: *p* = 0.0076–0.0464, Bonferroni's multiple comparison test). The fEPSP amplitude in the layer V of M1 region of the CoQ_10_-supplemented middle-aged mice and that of the age-matched controls showed a significant interaction and a significant difference between mice with and without CoQ_10_ supplementation (Fig. 3b left; the interaction between stimulus intensity and supplementation *p* < 0.0001,* F* (6, 228) = 5.064; the main effect of supplementation* p* = 0.0127, *F* (1, 38) = 6.843, two-way repeated-measures ANOVA). On the other hand, there was no significant interaction or difference in the fEPSP amplitude in the layer V of M2 region between mice with and without CoQ_10_ supplementation (Fig. 3b right; the interaction between stimulus intensity and supplementation *p* = 0.9032, *F* (6, 240) = 0.3607; the main effect of supplementation* p* = 0.9095,* F* (1, 40) = 0.0131, two-way repeated-measures ANOVA). In summary, the connection between layers II/III and layer V in the mouse motor cortex showed an age-related decline in fEPSP amplitude in the layer V of M1 region, which was restored by CoQ_10_ supplementation by drinking water for 1 week. There was no significant interaction or difference in the fEPSP amplitude in the layer V of M1 region between young adult mice without CoQ_10_ supplementation and middle-aged mice with CoQ_10_ supplementation (Data shown in Fig. 3a left and 3b left; the interaction between stimulus intensity and age *p* = 0.5174, *F* (6, 138) = 0.8719; the main effect of age* p* = 0.8316,* F* (1, 23) = 0.0463, two-way repeated-measures ANOVA).

### CoQ_10_ supplementation did not affect short-term plasticity in the motor cortex

**Figure 4:**
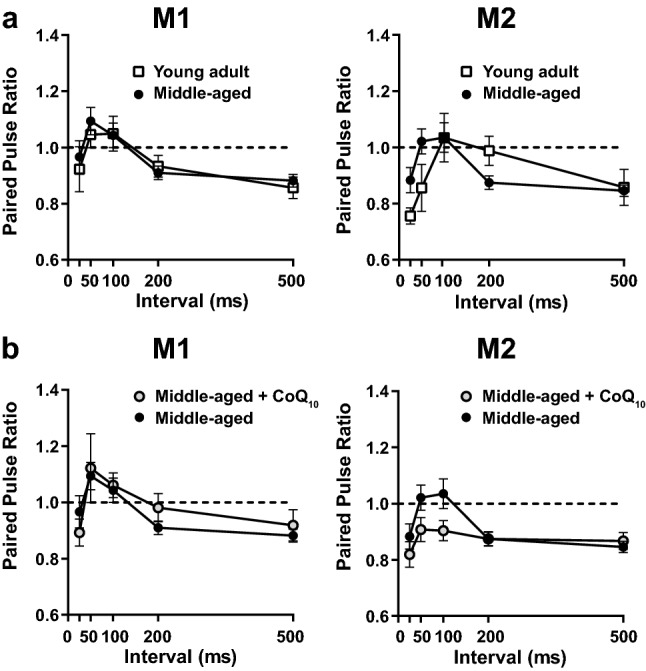
Short-term plasticity in the motor cortex was not affected by age or CoQ_10_ supplementation. (**a**) Paired pulse ratios (PPRs) at various stimulus intervals (25, 50, 100, 200, and 500 ms) were comparable in the M1 region (young adult, *n* = 14 slices from 5 mice; middle-aged, *n* = 27 slices from 9 mice) and the M2 region (young adult, *n* = 9 slices from 4 mice; middle-aged, *n* = 24 slices from 10 mice; no significant difference by age). (**b**) CoQ_10_ supplementation did not alter the PPRs in the M1 or M2 regions of middle-aged mice compared to those of the age-matched controls (M1: middle-aged + CoQ_10_, *n* = 8 slices from 4 mice; middle-aged, *n* = 27 slices from 9 mice; M2: middle-aged + CoQ_10_, *n* = 12 slices from 5 mice; middle-aged, *n* = 24 slices from 10 mice; no significant differences with supplementation). The middle-aged control data in (**b**) are identical to those in (**a**). Values are expressed as the mean ± SEM of independent experimental groups. Statistical analyses were performed using two-way repeated-measures ANOVA. For details of the data, see Supplementary Table 3.

We assessed short-term synaptic plasticity of the major intralaminar excitatory connection from layers II/III to layer V in the motor cortex^22,28^ using cortical slices and measuring paired-pulse ratios (PPRs) following paired stimulation at 25- to 500-ms intervals. There was no significant interaction or main effect of age group and stimulus interval on PPRs in the M1 region (Fig. 4a left; the interaction between interval and age *p* = 0.8607, *F* (4, 156) = 0.3252; the main effect of age *p* = 0.7295, *F* (1, 39) = 0.1213, two-way repeated-measures ANOVA). In the M2 region, there was a significant interaction between age group and stimulus interval but there was not a significant main effect of age, and Bonferroni's multiple comparison showed no significant differences in PPRs between young adult and middle-aged mice among all stimulus intervals (Fig. 4a right; the interaction between interval and age *p* = 0.0293, *F* (4, 124) = 2.790; the main effect of age *p* = 0.4784, *F* (1, 31) = 0.5150, two-way repeated-measures ANOVA). In addition, there were no significant interactions or differences in PPRs in the M1 and M2 regions of middle-aged mice with and without CoQ_10_ supplementation for 1 week (Fig. 4b; M1, the interaction between interval and supplementation *p* = 0.6793, *F* (4, 132) = 0.5777; the main effect of supplementation *p* = 0.7833, *F* (1, 33) = 0.0769; M2, the interaction between interval and supplementation *p* = 0.1694, *F* (4, 136) = 1.633; the main effect of supplementation *p* = 0.1640,* F* (1, 34) = 2.023, two-way repeated-measures ANOVA). These results suggested that physiological aging and CoQ_10_ supplementation did not affect the short-term plasticity of the layers II/III to V connection in the motor cortex in these preparations.

### Acute CoQ_10_ treatment induced NMDA receptor-dependent LTP

Mitochondria have effects on age-related synaptic plasticity^29^. Therefore, we studied the involvement of plasticity enhancement as a mechanism that augmented the fEPSP amplitude in the motor cortex of CoQ_10_-supplemented middle-aged mice. A larger fEPSP amplitude is recorded after long-term potentiation (LTP) induction^30,31^, and LTP can be induced in the motor cortex by motor-skill learning or several induction methods^30,32^. Furthermore, a larger fEPSP amplitude can be retained for months in the motor cortex after motor-skill learning^33^.

**Figure 5:**
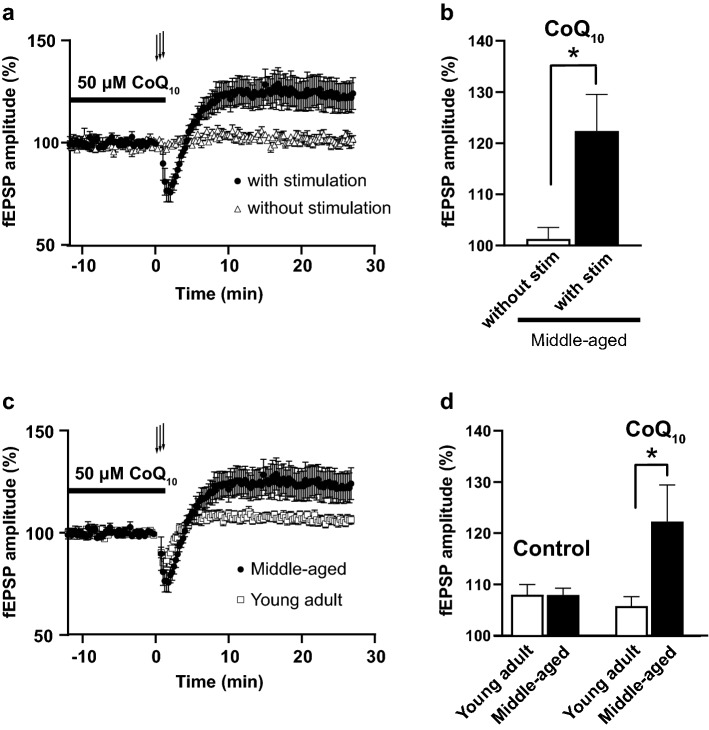
Acute CoQ_10_ administration enhanced LTP in the motor cortex of middle-aged mice. The fEPSP amplitude in the layer V of M1 region was recorded with a single glass electrode. The stimulation electrode was placed in layers II/III as described in the methods section. (**a**) The fEPSP amplitude increased compared to baseline amplitude in slices treated acutely with CoQ_10_ and high-frequency stimulation but not without high-frequency stimulation. The graph shows plots of the mean normalized fEPSP amplitude recorded in slices prepared from middle-aged mice with CoQ_10_ (without stim, *n* = 5 slices from 4 middle-aged mice; with stim, *n* = 18 slices from 10 mice). fEPSP amplitudes were normalized to baseline amplitudes before stimulation. The horizontal lines indicate the time of CoQ_10_ administration (50 μM, 20–25 min), and the arrows indicate the timing of the high-frequency stimulation (three trains of 100 pulses at 100 Hz applied at 15-s intervals). (**b**) The mean normalized fEPSP amplitude at 25–27 min after CoQ_10_ administration with and without high-frequency stimulation (*p < 0.05, Welch's *t* test). (**c**) Different magnitudes of LTP were induced in slices prepared from young adult and middle-aged mice treated acutely with CoQ_10_ and high-frequency stimulation. The plots of normalized fEPSP amplitudes are shown as in (**a**). (**d**) The mean normalized fEPSP amplitudes at 25–27 min after high-frequency stimulation with and without CoQ_10_ administration (control: young adult,* n* = 20 slices from 10 mice; middle-aged, *n* = 16 slices from 9 mice; CoQ_10_: young adult, *n* = 19 slices from 4 mice; middle-aged,* n* = 18 slices from 10 mice; young adult with CoQ_10_ compared to middle-aged with CoQ_10_; *p < 0.05, two-way ANOVA with Bonferroni's multiple comparison test). The high-frequency stimulation induced slight LTP (105–108%) in slices of young adult mice with and without CoQ_10_ administration and middle-aged mice without CoQ_10_ administration. Acute CoQ_10_ administration enhanced the magnitude of LTP on average by 22.39%. The middle-aged with CoQ_10_ data in (**d**) are identical to those in (**b**). The control and experimental groups had different numbers of mice because few experiments were discarded due to the baseline variation being greater than 10% in the first 20 min of recording. Values are expressed as the mean ± SEM of independent experimental groups. For details of the data, see Supplementary Table 3.

We tested whether acute CoQ_10_ administration (50 μM) to brain slices could enhance fEPSP amplitude in the connection between layers II/III and layer V in the motor cortex. Figure 5a shows normalized fEPSP amplitudes in the presence of CoQ_10_ and during CoQ_10_ washout. Acute CoQ_10_ administration alone did not augment fEPSP amplitude in the layer V of M1 region after the treatment (Fig. 5a; middle-aged without stimulation, averaged fEPSP, between − 2 and 0 min and 25–27 min *p* = 0.5931,* t* (4) = 0.5799, paired *t* test). The combination of CoQ_10_ administration (50 μM, 20–25 min before and during LTP induction) and high-frequency stimulation induced an LTP of 122.39 ± 7.15% of baseline in middle-aged mice (Fig. 5a; middle-aged with stimulation, averaged fEPSP, between − 2 and 0 min and 25–27 min *p* = 0.0061, *t* (17) = 3.133, paired *t* test). The mean normalized fEPSP amplitude at 25–27 min after CoQ_10_ administration was significantly higher with high-frequency stimulation than without stimulation (Fig. 5b, without stimulation, *n* = 5; with stimulation, *n* = 18; *p* = 0.0107,* t* (19.67) = 2.819, Welch's *t* test).

Next, we evaluated the magnitude of LTP in slices taken from young adult and middle-aged mice by measuring changes in normalized fEPSP amplitude before and after LTP induction with high-frequency stimulation with and without acute CoQ_10_ administration. High-frequency stimulation with acute CoQ_10_ administration induced LTP in slices of young adult and middle-aged mice (Fig. 5c; CoQ_10_: young adult, *p* = 0.0051, *t* (18) = 3.187; middle-aged, *p* = 0.0061,* t* (17) = 3.133, paired *t* test). The high-frequency stimulation without CoQ_10_ administration induced LTP in slices of young adult and middle-aged mice similarly (Fig. 5d; Control, young adult: 108.1 ± 1.98% of baseline, *p* = 0.0006, *t* (19) = 4.092; Middle-aged: 108.03 ± 1.35% of baseline, *p* < 0.0001, *t* (15) = 5.962, paired *t* test). The fEPSP amplitude in slices of young adult and middle-aged mice after LTP induction with or without acute CoQ_10_ administration showed a significant interaction and a significant difference of age by two-way ANOVA. The magnitude of LTP was significantly greater in CoQ_10_-treated slices of middle-aged mice than CoQ_10_-treated slices of young adult mice (Fig. 5d; the interaction between treatment and age *p* = 0.0355, *F* (1, 69) = 4.598; the main effect of treatment *p* = 0.0371,* F* (1, 69) = 4.519, two-way ANOVA; young adult CoQ_10_ compared to middle-aged CoQ_10_, *p* = 0.0195, Bonferroni’s multiple comparison test). These results suggested that exogenous CoQ_10_ and increased neuronal activity enhanced the synaptic plasticity efficacy of middle-aged mice.

**Figure 6:**
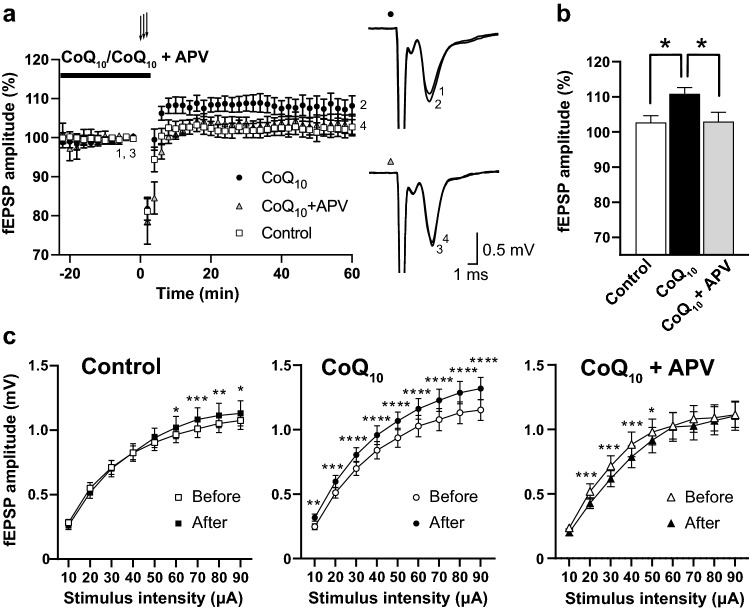
CoQ_10_-dependent LTP was blocked by an NMDA receptor antagonist and increased the basal fEPSP amplitudes. (**a**) The graph shows the averaged time course of the normalized fEPSP amplitude recorded in layer V in slices prepared from middle-aged mice with high-frequency stimulation alone (control), in the presence of CoQ_10_, and in the presence of CoQ_10_ with APV (each *n* = 11 slices from 11 mice). The ordinates represent normalized fEPSP amplitude, where 100% corresponds to the averaged amplitude recorded before high-frequency stimulation, and the abscissa represents the time of recording. The horizontal line above the plots indicates the time of drug application. The arrows indicate the timing of the high-frequency stimulation (three trains of 100 pulses at 100 Hz applied at 15-s intervals). The inserts on the right show traces from representative recordings. Each trace is the average of 2 min immediately before the high-frequency stimulation (1, CoQ_10_; 3, CoQ_10_ + APV) and 2 min at the 58- to 60-min time point (2, CoQ_10_; 4, CoQ_10_ + APV). (**b**) The blockage of NMDA receptors with APV (50 μM, 23–25 min before and during LTP induction) in the presence of CoQ_10_ occluded the LTP induction in the layer V of M1 region of the middle-aged mice (control, CoQ_10_, CoQ_10_ + APV, each *n* = 11, *p < 0.05, one-way ANOVA with Bonferroni's multiple comparison test). (**c**) Average fEPSP amplitudes before (before) and 1 h after (after) the high-frequency stimulation recorded in ACSF in a range from 10 to 90 μA current stimuli (each *n* = 11 slices from 11 mice). Statistical analyses were performed using two-way repeated-measures ANOVA with Bonferroni's multiple comparisons for the control group, the CoQ_10_ group, and the CoQ_10_ + APV group (****p < 0.0001; ***p < 0.001; **p < 0.01; *p < 0.05). Values are the mean ± SEM of independent experimental groups. For details of the data, see Supplementary Table 3.

An age-related increase in NMDA receptor-dependent LTP has been observed in rat hippocampal slices^34^. Therefore, the NMDA receptor selective antagonist 2-amino-5-phosphonovaleric acid (APV) was applied with CoQ_10_ during LTP induction in the motor cortex to examine the role of NMDA receptors in the age-related LTP induction described in Fig. 5. The high-frequency stimulation in the presence of CoQ_10_ (50 μM, 23–25 min before and during LTP induction) induced an LTP of on average 110.88 ± 1.77% of baseline in the M1 region of middle-aged mice (Fig. 6a, CoQ_10_; averaged fEPSP, between − 2 to 0 min and 58 to 60 min *p* = 0.0001, *t* (10) = 6.139, paired *t* test). However, the high-frequency stimulation in the presence of CoQ_10_ and APV (each 50 μM, 23–25 min before and during LTP induction) failed to induce LTP in slices taken from the same mice (Fig. 6a, CoQ_10_ + APV: 102.94 ± 2.65% of baseline; averaged fEPSP, between − 2 to 0 min and 58 to 60 min* p* = 0.2924, *t* (10) = 1.111, paired *t* test). APV significantly blocked CoQ_10_-dependent LTP induction to a level similar to that of the control (Fig. 6b; *p* = 0.0167, *F* (2, 30) = 4.703, one-way ANOVA; control compared to CoQ_10_, *p* = 0.0343; CoQ_10_ compared to CoQ_10_ + APV, *p* = 0.0414, Bonferroni's multiple comparison test). These results suggested that CoQ_10_-dependent LTP of the M1 region in middle-aged mice was dependent on NMDA receptors.

### Acute CoQ_10_ treatment augmented basal fEPSP amplitude

We hypothesized that CoQ_10_-dependent LTP might be part of the mechanism augmenting the basal fEPSP amplitude in middle-aged mice supplemented with CoQ_10_ by drinking water. Figure 6c shows the average amplitude of fEPSPs recorded from 5 trials of each current stimulus in 1 brain slice before the LTP experiment and 1 h after the high-frequency stimulation shown in Fig. 6a, b. In the control condition, there was no significant main effect between the fEPSP amplitudes before/after the high-frequency stimulation, but there was a significant interaction between the fEPSP amplitudes before/after stimulation and the stimulus intensity (the interaction between the fEPSP amplitudes before/after stimulation and the stimulus intensity *p* < 0.0001,* F* (8, 80) = 5.145; the main effect of the fEPSP amplitudes before/after stimulation *p* = 0.4513, *F* (1, 10) = 0.6145, the main effect of the stimulus intensity *p* < 0.0001, *F* (8, 80) = 102.2; two-way repeated-measures ANOVA). Bonferroni's multiple comparison showed significant differences between the fEPSP amplitudes before/after the high-frequency stimulation among stimulus intensities between 60 and 90 μA (Fig. 6c left; Before compared to After, 60–90 μA: *p* = 0.001 to 0.0279). However, when CoQ_10_-dependent LTP expression was observed, there was a significant interaction between the fEPSP amplitudes before/after stimulation and the stimulus intensity and a significant difference between the fEPSP amplitudes before/after stimulation. The fEPSP amplitudes increased significantly at an average of 115.95 ± 1.61% between the two recording time points (Fig. 6c center; the interaction between the fEPSP amplitudes before/after stimulation and the stimulus intensity *p* = 0.0067,* F* (8, 80) = 2.912; the main effect of the fEPSP amplitudes before/after stimulation *p* = 0.0031, *F* (1, 10) = 14.92, two-way repeated-measures ANOVA; Before compared to After, 10–90 μA: *p* < 0.0001 to *p* = 0.0031, Bonferroni's multiple comparison test). In contrast, when coadministration of CoQ_10_ and APV occluded LTP expression, there was a significant interaction between the fEPSP amplitudes before/after stimulation and the stimulus intensity by two-way repeated-measures ANOVA and a significant difference between the fEPSP amplitudes before/after stimulation. The fEPSP amplitudes were significantly smaller among 20–50 μA stimuli (Fig. 6c right; the interaction between the fEPSP amplitudes before/after stimulation and the stimulus intensity* p* = 0.0057, *F* (8, 80) = 2.980; the main effect of fEPSP amplitudes before/after stimulation* p* = 0.0076, *F* (1, 10) = 11.09, the main effect of the stimulus intensity *p* < 0.0001, *F* (8, 80) = 88.42; two-way repeated-measures ANOVA; Before compared to After, 20–50 μA: *p* = 0.0001 to 0.0359, Bonferroni's multiple comparison test). These results suggested that basal fEPSP amplitudes were augmented when LTP expression was observed; therefore, CoQ_10_-dependent LTP may have improved the fEPSP amplitudes of the M1 region motor cortex of the CoQ_10_-supplemented middle-aged mice.

## Discussion

The middle-aged mice showed an age-related decline in motor function (Fig. 1a, c). Concomitantly, the M1 region of the middle-aged mice showed an age-related decline in fEPSP amplitude in the pathway from layers II/III to layer V neurons (Fig. 3a). The decreased motor function and fEPSP amplitude were reverted to the young adult level by supplementing CoQ_10_ by drinking water for 1 week (Figs. 1a, 3b). Furthermore, acute CoQ_10_ treatment of brain slices induced LTP in the layer V of M1 region of middle-aged mice (Fig. 5a, c). This LTP induction depended on exogenous CoQ_10_, high-frequency stimulation, and NMDA receptors; however, acute CoQ_10_ administration alone did not alter the fEPSP amplitude (Fig. 5a). Coadministration of CoQ_10_ and APV reduced basal synaptic transmission (Fig. 6c, right), which also indicates the contribution of NMDA receptors in the pathway from layers II/III to layer V neurons at LTP induction. These results suggested that a change in the efficacy of plasticity may be the underlying mechanism for the fEPSP amplitude recovery by CoQ_10_ treatment. Indeed, we demonstrated that CoQ_10_-dependent LTP in the layer V of M1 region translates to enhanced fEPSP amplitude (Fig. 6c). To our knowledge, this report is the first to demonstrate that the pathway from layers II/III to V of the M1 region shows (a) an age-related decrease in fEPSP amplitude and (b) LTP in middle-aged mice. We identified an age-related alteration and CoQ_10_ and NMDA receptor dependency of LTP induction in the M1 region.

The efficacy of CoQ_10_ depends on its formulation^35^. Nanoformulations of CoQ_10_ have higher bioavailability than regular CoQ_10_ and have been reported to increase brain CoQ_10_ content and protect neurons by oral administration^7,36^. A previous study by Takahashi et al.^1^ used the water-soluble nanoformula product of Nisshin Pharma (Aqua Q_10_L10). To test whether the beneficial effect of CoQ_10_ supplementation could be generalized, we used a water-soluble nanoformula-type CoQ_10_ from Petroeuroasia (40SP) in the behavioral and OCR analyses of this study. CoQ_10_ (40SP) showed a beneficial effect on motor function and the oxygen consumption rate of brain mitochondria in middle-aged mice, similar to Aqua Q_10_L10. These results demonstrated that the beneficial effect of CoQ_10_ supplementation could be confirmed in water-soluble nanoformula-type CoQ_10_ from at least two different sources and suggested that the beneficial effect of CoQ_10_ could be generalized.

Elderly individuals suffer a progressive loss of muscle mass and strength (sarcopenia) and motor function^37–41^. However, the motor deficit in middle-aged mice is less likely to be due to motor neuron loss, NMJ denervation, or muscle atrophy. NMJ denervation is not detected significantly at or earlier than 18 months of age in mice^42,43^. Similarly, the maintenance of NMJ number suggests that spinal motor neurons are preserved in middle-aged mice^44^. A decline in muscle contractility is less prominent earlier than 20 months of age in mice^44^, and we also confirmed that muscle strength did not change significantly with CoQ_10_ supplementation (Fig. 1b, d).

Age-related changes in electrophysiological activity in layer V have been linked to motor function deficits in humans and animals. Middle-aged humans (between the late 50 s and early 60 s) showed more intracortical inhibition and less intracortical facilitation in the motor cortex than young adults when examined using transcranial magnetic stimulation^45^. Elderly individuals in their 70 s exhibited similar but more profound intracortical inhibition and less intracortical facilitation^46^. These data suggested an age-related decline in neuronal activity in the motor cortex of humans due to an altered balance of excitatory and inhibitory circuits. The correlation of hypoexcitability in the motor cortex and behavioral defects has also been implicated in chronic obstructive pulmonary disease (COPD) and amyotrophic lateral sclerosis (ALS) patients ^47,48^. In contrast, motor function improved when the activity of motor cortex layer V neurons was increased using optogenetic stimulation in Parkinson's disease model mice^49^. Layer V pyramidal neurons directly evoke or control the rhythm of whisker movements in rodents^50^. These observations suggest that the excitability level of layer V neurons of the motor cortex is important to maintain motor functions.

Neuronal plasticity enhancers augment motor-skill learning or accelerate rehabilitation after brain damage^51,52^. In the rat motor cortex, the LTP-like plasticity of M1 region augments motor-skill learning and rehabilitation effects^53^. LTP can be induced in the motor cortex by motor-skill learning^32,33^, and a larger fEPSP amplitude can be stabilized for months in the motor cortex after motor-skill learning^33^. LTP is also naturally induced by the environment or sensory stimuli. Enriched environmental exposure changed cellular excitability and synaptic transmission, induced NMDA-dependent LTP^54,55^ and enhanced learning^56^. Sensory stimulation, such as rhythmic stimulation of whiskers, also induced NMDA-dependent LTP^57,58^. An age-related increase in NMDA receptor-dependent LTP has been observed in rat hippocampal slices^34^. These types of LTP may have been induced in the middle-aged mice supplemented with CoQ_10_ by drinking water and contributed to recovering fEPSP amplitude and motor function.

CoQ_10_ supplementation by drinking water in middle-aged mice enhanced complex I activity in the brain mitochondria (Fig. 2). Considering that oxidative phosphorylation is associated with oxidative stress^59,60^, CoQ_10_ supplementation might induce higher oxidative stress, and excessive oxidative stress impairs cognitive function^61,62^. However, CoQ_10_ supplementation by drinking water reduces oxidative stress and improves cognitive function^63^. Furthermore, exogenous CoQ_10_ administration is protective against age-related and pathological oxidative stress^64,65^. Therefore, the antioxidant status in the brain of the CoQ_10_-supplemented middle-aged mice may be beneficial overall for the behavioral outcome of these mice in the current study.

CoQ_10_ supplementation had a beneficial effect on the motor function of the middle-aged mice and rescued their behavior to the young adult level (Fig. 1a). The beneficial effect of CoQ_10_ was partially achieved by enhancing the excitability level of layer V neurons in the M1 region. We hypothesized that the basal fEPSP amplitude level was enhanced in the middle-aged mice during CoQ_10_ supplementation (Fig. 3) by the continuation and retention of LTP-like plasticity^33,66^, such as CoQ_10_-dependent LTP (Figs. 5, 6). The enhanced LTP induction efficacy augments the rehabilitation-like effect to improve the pole test latency (Fig. 1a, b). These effects might be similar to the recovery of motor function after stroke and nervous system damage by rehabilitation training, which makes use of the plasticity and recovery function of the central nervous system. Therefore, these results suggest the possibility of translational application of CoQ_10_ supplementation in the following circumstances: (1) oral CoQ_10_ administration as preventive care for age-related motor decline and (2) the enhancement of plasticity of the primary motor cortex to improve motor function in elderly individuals.

## Methods

### Animals

All experimental procedures were approved by the Animal Care and Use Committee of the Tokyo Metropolitan Institute for Geriatrics and Gerontology. All experiments were carried out in accordance with the approved animal care and use protocol and the Guidelines for Care and Use of Laboratory Animals. The authors complied with the ARRIVE 2.0 guidelines^67,68^. C57BL/6NCr male mice were purchased from Japan SLC Inc. (Shizuoka, Japan) at 4 weeks old. The mice were housed in groups of two to five per cage and maintained in a temperature- and humidity-controlled environment with a 12-h light/dark cycle. We used a total of 56 young adult mice (6–8 months old) and 86 middle-aged mice (15–18 months old) given ad libitum food and water. For details of animal numbers, see Supplementary Table 1.

### CoQ_10_-supplemented mouse experiments

**Figure 7:**
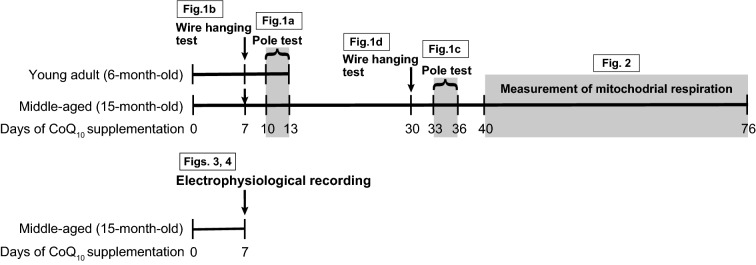
Schematic diagram of the experimental design for CoQ_10_-supplemented mice. Mice were supplemented with CoQ_10_ by drinking water for at least 1 week before starting the experiment. Age-matched control mice were given normal drinking water. The mice were evaluated using the wire hanging test at 7 days after starting CoQ_10_ supplementation for the young adult and middle-aged groups and again at 30 days for the middle-aged group. The pole test was performed 10–13 days after starting the supplementation for the young adult and middle-aged groups and again at 33–36 days for the middle-aged group. The brain mitochondrial respiration rates were measured between 40 and 76 days after starting CoQ_10_ supplementation. Therefore, the mice were 15 months old at the beginning and approximately 17 months old at the completion of the experiment. Electrophysiological recording with a multi-electrode array was performed using 15-month-old mice supplemented with CoQ_10_ by drinking water for 7 days. Figure numbers indicate the relative timing of corresponding experiments.

CoQ_10_-supplemented mice were analyzed by behavioral experiments, measurement of brain mitochondrial respiration, and electrophysiological recording with a multi-electrode array. Drinking water containing 150 μM water-soluble nanoformula-type CoQ_10_ (Figs. 1, 2, Coenzyme Q_10_ 40% Water-dispersive Powder 40SP, Petroeuroasia Co. Ltd., Shizuoka, Japan; Figs. 3, 4, Aqua Q_10_L10-NF, Nisshin Pharma Inc., Tokyo, Japan) were prepared in light-protected bottles twice weekly and given ad libitum to mice until sacrifice based on the preceding studies^1,17^. An overview of the mouse experiments is shown in Fig. 7.

### Behavioral experiments

We performed a priori power analysis using G-Power software^69^ to estimate the required sample size for behavioral experiments. The experiments aimed to analyze the effects of aging and CoQ_10_ supplementation with 95% actual power using a two-way analysis of variance (ANOVA) with four groups, a significance level of *p* < 0.05 and an effect size of f = 0.4. The required total sample size was 84 mice, 21 mice per group. We decided to use 20 mice in each group and house five mice per cage for social housing. The middle-aged mouse group had one less data point due to death (Fig. 1c, d). Mice were handled by the experimenter for three consecutive days for habituation and then sequentially tested on the wire hanging and pole tests. The behavior tests were performed by personnel blinded to the treatment group and the animals were randomized.

#### Wire hanging test

The four-limb wire hanging test (O'Hara & Co., Ltd., Tokyo, Japan) was performed as described previously^70^. The latency to fall from the grid was recorded from two trials with a 30 min intertrial interval. The longer latency was considered the representative value for the mice.

#### Pole test

The pole test was first designed to evaluate bradykinesia in a Parkinson's disease murine model and has been used to measure motor coordination deficits^18–20^. Initially, mice were habituated to an experimental cage and the pole (length 45 cm, diameter 1 cm). The day before the test, four training trials were conducted. During the test, the time required for mice to turn their body and feet completely downward (T-turn) and the total time to descend to the floor of the experimental cage (T-total) were measured with 15 min intertrial intervals. The average of five test trials was used as the representative value.

### Measurement of mitochondrial respiration

After the behavioral experiments (16 months old; control, *n* = 20; CoQ_10_, *n* = 19), mitochondrial fractions from one brain hemisphere were isolated as previously described^17^. We measured NADH-linked (Complex I) respiration of brain mitochondrial fractions, which declined with age^1^. The OCRs of mitochondrial fractions in mitochondrial respiration medium (MiR05; 0.5 mM EGTA, 3 mM MgCl2, 60 mM lactobionic acid, 20 mM taurine, 10 mM KH_2_PO_4_, 20 mM HEPES, 110 mM D-sucrose, 1 g/l bovine serum albumin; pH 7.1) containing 10 mM cytochrome c were determined at 37 °C using high-resolution respirometry (Oxygraph-2k; Oroboros Instruments, Innsbruck, Austria). NADH-linked respiration was assessed by adding 2.5 mM ADP in the presence of 5 mM malate and 10 mM glutamate, as previously described^17,71^. The data were normalized to total protein of the mitochondrial fraction in the high-resolution respirometry chamber (1.23–2.00 mg of protein). The respiration rates were analyzed by pairing the control and CoQ_10_-supplemented groups in each measurement by personnel blinded to the treatment group. Three samples in the control group were not measurable due to insufficient sample volume caused by a human error. Two samples in the CoQ_10_ group were not measurable due to an equipment failure of the Oxygraph-2k.

### Brain slice preparation for electrophysiology

Mouse brains were cut at a 15°–20° angle inclined rostrally against the coronal plane of the cortex yielding slices with apical dendrites of layer V neurons parallel to the cut surface^22^. Brains were cut into 300 μm thick slices using a Pro 7 Linear Microslicer (Dosaka, Kyoto, Japan) in chilled artificial cerebrospinal fluid (ACSF, 124 mM NaCl, 3 mM KCl, 1 mM NaH_2_PO_4_, 1.2 mM MgCl_2_, 2.4 mM CaCl_2_, and 10 mM glucose) with 26 mM NaHCO_3_ bubbled with 95% O_2_ and 5% CO_2_ for oxygenation and pH adjustment to pH 7.4. The slices were incubated in 30 °C ACSF for 1 h for recovery and then maintained in room temperature (23–25 °C) ACSF until recordings. We selected a small but empirically adequate sample size for all electrophysiological experiments because this was the first evaluation.

### Multi-electrode array recording

We separately analyzed the primary motor (M1) and secondary motor (M2) cortices in the motor cortex (approximately + 0.8 to + 1.2 mm from bregma based on the mouse brain atlas by Paxinos and Franklin^72^). Evoked field excitatory postsynaptic potentials (fEPSPs) were recorded with a multi-electrode array (60pMEA200/30iR-Ti; Multi Channel Systems, Reutlingen, Germany) at room temperature by placing the multiple electrodes in the layer V of M1 or M2 regions. A stimulating glass electrode filled with 1 M NaCl (resistance < 1 MΩ) was placed in layers II/III of the motor cortex. Signals were sampled at room temperature at 50 kHz using a multi-electrode array 1060 amplifier with a band pass filter (3 kHz) (Multi Channel Systems), digitized with a Digidata 1440 series acquisition interface (Molecular Devices, San Jose, USA), and analyzed with pCLAMP10 software (Molecular Devices). Among the electrodes in layer V, we analyzed data from one electrode that recorded the largest fEPSP amplitude at 80 µA current stimuli. A paired-pulse ratio (PPR) was calculated as the ratio of fEPSPs (second amplitude/first amplitude) recorded during paired stimulation (60 μA) in 25- to 500-ms intervals. At the end of the recordings, an AMPA and kainate receptor antagonist, 6-cyano-7-nitroquinoxa-line-2,3-dione (CNQX, 10 μM), and an NMDA receptor antagonist, 2-amino-5-phosphonovaleric acid (APV, 25 μM), were bath-applied to block synaptic transmission and to confirm the disappearance of fEPSPs (data not shown). We used a total of 20 mice (young adult: *n* = 5; middle-aged control: *n* = 10; middle-aged supplemented with CoQ_10_: *n* = 5). The recordings were performed on brain slices in randomized order.

### Single glass electrode recording

Evoked fEPSPs were recorded at 30 °C with a borosilicate glass electrode (3.0–4.5 MΩ resistance) filled with ACSF and placed in the layer V of M1 region. A stimulating glass electrode filled with 1 M NaCl was placed in layers II/III of the M1 region in the radial direction from the recording electrode. Signals were sampled at 10 kHz and filtered at 1 kHz using an EPC 10 amplifier (HEKA Elektronik, Lambrecht/Pfalz, Germany) and analyzed offline with FITMASTER software (version 2 × 90.2, HEKA Elektronik). Baseline fEPSPs were evoked with short pulses (100 μs at 0.067 Hz) and recorded for at least 10 min preceding CoQ_10_ administration. The stimulus intensity was adjusted to a level where 65–80% of the maximal fEPSP amplitude was evoked. We used slices prepared from the same animals and recorded using alternating stimulus and pharmacological treatments on the same day. The experiment was discarded if the baseline variation was greater than 10% in the first 20 min of recording. LTP was induced with three trains of high-frequency stimulation consisting of 100 pulses at 100 Hz applied at 15-s intervals. The magnitude of LTP was expressed as the % change in the average fEPSP amplitude obtained from 25 to 27 min (Fig. 5) or 58 to 60 min (Fig. 6) after LTP induction to the average amplitude of baseline fEPSP measured during the 2 min before the high-frequency stimulation. We used a total of 26 mice (young adult: *n* = 11; middle-aged: *n* = 15) in Fig. 5.

For the APV experiments, water-soluble nanoformula-type CoQ_10_ and APV (each 50 μM) dissolved in ACSF were bath-applied to the chamber from 23 to 25 min before LTP induction and then washed out after high-frequency stimulation. Immediately before and after the LTP experiments, the input‒output relationship was examined by varying the stimulus intensity. Three conditions (ACSF, CoQ_10_, CoQ_10_ + APV) were tested in each mouse, and data from mice that showed more than 5% CoQ_10_-dependent LTP were analyzed (Fig. 6, 11 mice were analyzed among 16 mice tested at 15–18 months old with the same birthdate). The recordings were performed on brain slices prepared from the same animals and treated in randomized order with three experimental conditions.

### Drugs

APV was purchased from Tocris Bioscience (Bristol, UK). All other drugs were purchased from Sigma‒Aldrich (St. Louis, USA).

### Statistics

Statistical differences of three or more groups were assessed using one-way analysis of variance (ANOVA) or two-way repeated-measures ANOVA with a multiple comparison test with Bonferroni's correction (Prism version 8.4.3, GraphPad Software, La Jolla, USA). Statistical differences between the two groups or conditions were assessed using the two-tailed Welch's *t* test or paired *t* test. All values are expressed as the mean ± SEM. Statistical significance was set at *p* < 0.05. For details of the statistical analyses, see Supplementary Table 2.

## Supplementary Information

Supplementary Information.

## Data Availability

The datasets generated in this study are available from the corresponding author upon reasonable request.
