# Supplementary material for: Coenzyme Q10 supplementation improves the motor function of middle-aged mice by restoring the neuronal activity of the motor cortex
Source: Sci Rep. 2023 Mar 15;13:4323. doi: 10.1038/s41598-023-31510-1 (PMC10017826; doi:10.1038/s41598-023-31510-1)
Supplement: Supplementary file 1 — Supplementary Information. [file 41598_2023_31510_MOESM1_ESM.pdf]

Supplementary Tables

Title:

Coenzyme Q_{10} supplementation improves the motor function of middle-aged mice by restoring the neuronal activity of the motor cortex

Authors:

Ritsuko Inoue[1,*], Masami Miura[1,3], Shuichi Yanai[2], Hiroshi Nishimune[1,4,*]

Affiliation:

[1]Laboratory of Neurobiology of Aging, [2] Laboratory of Memory Neuroscience, Tokyo Metropolitan Institute for Geriatrics and Gerontology, Itabashi-ku, Tokyo 173-0015 Japan

[3]Saitama Central Hospital, Iruma-gun, Saitama 354-0045 Japan

[4]Department of Applied Biological Science, Tokyo University of Agriculture and Technology, 3-8-1 Harumicho, Fuchu-shi, Tokyo 183-8538, Japan

*** Corresponding Authors:**

Ritsuko Inoue, Ph.D. and Hiroshi Nishimune, Ph.D.

Laboratory of Neurobiology of Aging

Tokyo Metropolitan Institute for Geriatrics and Gerontology

35-2 Sakaecho, Itabashi-ku, Tokyo 173-0015 Japan

Phone: 81-3-3964-3241

Email: inoritu@tmig.or.jp, nishimun@tmig.or.jp

Supplementary Table 1. Summary of animal numbers

Number of animals per experiment							Sub-total number of animals per experiment	
							Young adult mice	Middle-aged mice
	Young adult mice Control	Young adult mice CoQ_{10}-supplemented	Middle-aged mice Control	Middle-aged mice CoQ_{10}-supplemented				
Fig.1a, b	20	20	20	20				
Fig.1c, d			↑	↑ (19*)				
Fig. 2			↑ (17**)	↑ (17**)				
**Total number of animals in Figs. 1, 2**	**20**	**20**	**20**	**20**			**40**	**40**
	Young adult mice		Middle-aged mice Control		Middle-aged mice CoQ_{10}-supplemented			
	M1	M2	M1	M2	M1	M2		
Fig. 3	5	←(4**)	10	←	5	←		
Fig. 4	↑	↑ (4**)	↑ (9**)	↑	↑ (4**)	↑ (4**)		
**Total number of animals in Figs. 3, 4**	**5**		**10**		**5**		**5**	**15**
			Middle-aged mice CoQ_{10}-treated w/o stim	Middle-aged mice CoQ_{10}-treated with stim				
Fig. 5a			4	10				
	Young adult mice Control	Young adult mice CoQ_{10}-treated	Middle-aged mice Control	Middle-aged mice CoQ_{10}-treated				
Fig. 5d	3	←	(8) →	↑				
	7	1	1					
**Total number of animals**		**11**	**5**	**10**			**11**	**15**
				Middle-aged mice CoQ_{10}-treated with stim				
Fig. 6			Number of animals not-analyzed as described in the methods section	5				
			Number of animals analyzed	11				
**Total number of animals**				**16**				**16**
						**Total number of animals**	**56**	**86**

*Reduced number of animals analyzed due to death

**Reduced number of animals analyzed as described in the methods section

Arrows indicate that the same animals were used as the column/row to which the arrow points.

Supplementary Table 2. Summary of statistical analyses

Figure number/Data	Analysis		Statistical value	
Fig.1a left	two-way ANOVA	Pole test, T-turn		
		interaction (supplementation x age)	F (1, 76) = 4.919	p = 0.0296
		main effect of supplementation	F (1, 76) = 11.95	p = 0.0009
		main effect of age	F (1, 76) = 25.77	p < 0.0001
	Bonferroni's multiple comparisons test	Young adult control vs. Middle-aged control	t (76.00) = 5.158	p < 0.0001
		Young adult control vs. Young adult CoQ_{10}	t (76.00) = 0.8759	p > 0.9999
		Young adult control vs. Middle-aged CoQ_{10}	t (76.00) = 1.146	p > 0.9999
		Middle-aged control vs. Young adult CoQ_{10}	t (76.00) = 6.034	p <0.0001
		Middle-aged control vs. Middle-aged CoQ_{10}	t (76.00) = 4.012	p = 0.0008
		Young adult CoQ_{10} vs. Middle-aged CoQ_{10}	t (76.00) = 2.022	p = 0.2805
Fig.1a right	two-way ANOVA	Pole test, T-total		
		interaction (supplementation x age)	F (1, 76) = 2.240	p = 0.1386
		main effect of supplementation	F (1, 76) = 8.686	p = 0.0043
		main effect of age	F (1, 76) = 1.015	p = 0.3168
Fig. 1b	two-way ANOVA	Wire hanging test		
		interaction (supplementation x age)	F (1, 76) = 0.0050	p = 0.9440
		main effect of supplementation	F (1, 76) = 0.0668	p = 0.7968
		main effect of age	F (1, 76) = 22.42	p < 0.0001
Fig. 1c	Welch's *t*-test	Pole test		
		T-turn	t (34.48) = 4.253	p = 0.0002
		T-total	t (36.93) = 3.875	p = 0.0004
Fig. 1d	Welch's *t*-test	Wire hanging test		
		Middle-aged control vs. Middle-aged CoQ_{10}	t (31.63) = 0.4741	p = 0.6387
Fig. 2	Welch's *t*-test	Brain mitochondrial respiration		
		Middle-aged control vs. Middle-aged CoQ_{10}	t (22.23) = 2.115	p = 0.0459

Fig. 3a left (M1)	two-way repeated measures ANOVA	interaction (stimulus intensity x age)	F (6, 246) = 1.257	p = 0.2778
		main effect of stimulus intensity	F (6, 246) = 69.49	p < 0.0001
		main effect of age	F (1, 41) = 5.572	p = 0.0231
Fig. 3a right (M2)	two-way repeated measures ANOVA	interaction (stimulus intensity x age)	F (6, 198) = 1.835	p = 0.0941
		main effect of stimulus intensity	F (6, 198) = 44.95	p < 0.0001
		main effect of age	F (1, 33) = 2.687	p = 0.1107
Fig. 3b left (M1)	two-way repeated measures ANOVA	interaction (stimulus intensity x supplementation)	F (6, 228) = 5.064	p < 0.0001
		main effect of stimulus intensity	F (6, 228) = 98.39	p < 0.0001
		main effect of supplementation	F (1, 38) = 6.843	p = 0.0127
	Bonferroni's multiple comparisons test	Middle-aged vs. Middle-aged + CoQ_{10}		
		Stimulus intensity: 20 μA	t (266.0) = 1.123	p > 0.9999
		Stimulus intensity: 30 μA	t (266.0) = 1.815	p = 0.4945
		Stimulus intensity: 40 μA	t (266.0) = 2.165	p = 0.2188
		Stimulus intensity: 50 μA	t (266.0) = 2.737	p = 0.0464
		Stimulus intensity: 60μA	t (266.0) = 3.071	p = 0.0165
		Stimulus intensity: 70 μA	t (266.0) = 3.077	p = 0.0162
		Stimulus intensity: 80 μA	t (266.0) = 3.305	p = 0.0076
Fig. 3b right (M2)	two-way repeated measures ANOVA	interaction (stimulus intensity x supplementation)	F (6, 240) = 0.3607	p = 0.9032
		main effect of stimulus intensity	F (6, 240) = 91.48	p < 0.0001
		main effect of supplementation	F (1, 40) = 0.0131	p = 0.9095
Young adult vs. Middle-aged + CoQ_{10} in Fig. 3a left, 3b left (M1)	two-way repeated measures ANOVA	interaction (stimulus intensity x age)	F (6, 138) = 0.8719	p = 0.5174
		main effect of stimulus intensity	F (6, 138) = 54.53	p < 0.0001
		main effect of age	F (1, 23) = 0.0463	p = 0.8316
Fig. 4a left (M1)	two-way repeated measures ANOVA	PPR		
		interaction (interval x age)	F (4, 156) = 0.3252	p = 0.8607
		main effect of interval	F (4, 156) = 9.699	p < 0.0001
		main effect of age	F (1, 39) = 0.1213	p = 0.7295
Fig. 4a right (M2)	two-way repeated measures ANOVA	PPR		
		interaction (interval x age)	F (4, 124) = 2.790	p = 0.0293

		main effect of interval	F (4, 124) = 6.208	p = 0.0001
		main effect of age	F (1, 31) = 0.5150	p = 0.4784
	Bonferroni's multiple comparisons test	Young adult vs. Middle-aged		
		interval: 25 ms	t (155.0) = 1.673	p = 0.4820
		interval: 50 ms	t (155.0) = 2.159	p = 0.1618
		interval: 100 ms	t (155.0) = 0.0023	p > 0.9999
		interval: 200 ms	t (155.0) = 1.481	p = 0.7034
		interval: 500 ms	t (155.0) = 0.1547	p > 0.9999
Fig. 4b left (M1)	two-way repeated measures ANOVA	PPR		
		interaction (interval x supplementation)	F (4, 132) = 0.5777	p = 0.6793
		main effect of interval	F (4, 132) = 6.197	p = 0.0001
		main effect of supplementation	F (1, 33) = 0.0769	p = 0.7833
Fig. 4b right (M2)	two-way repeated measures ANOVA	PPR		
		interaction (interval x supplementation)	F (4, 136) = 1.633	p = 0.1694
		main effect of interval	F (4, 136) = 4.988	p = 0.0009
		main effect of supplementation	F (1, 34) = 2.023	p = 0.1640
Middle-aged mice data shown in Fig. 5a	paired *t* - test	the % change in the fEPSP amplitude		
		with stimulation and CoQ_{10}, Middle-aged (averaged fEPSP amplitude, between -2 to 0 min and 25 to 27 min)	t (17) = 3.133	p = 0.0061
		without stimulation (averaged fEPSP amplitude, between -2 to 0 min and 25 to 27 min)	t (4) = 0.5799	p = 0.5931
Fig. 5b	Welch's *t* -test	fEPSP amplitude (%)		
		without stim vs. with stim	t (19.67) = 2.819	p = 0.0107
Young adult mice data shown in Fig. 5c	paired *t* - test	the % change in the fEPSP amplitude		
		CoQ_{10}, Young adult (averaged fEPSP amplitude, between -2 to 0 min and 25 to 27 min)	t (18) = 3.187	p = 0.0051
Remaining LTP data not illustrated fEPSP amplitude plots in Fig. 5a, c	paired *t* - test	the % change in the fEPSP amplitude		
		Control, Young adult (averaged fEPSP amplitude, between -2 to 0 min and 25 to 27 min)	t (19) = 4.092	p = 0.0006
		Control, Middle-aged (averaged fEPSP amplitude, between -2 to 0 min and 25 to 27 min)	t (15) = 5.962	p < 0.0001

Fig. 5d	two-way ANOVA	magnitude of LTP(%)		
		interaction (treatment x age)	$F (1, 69) = 4.598$	$p = 0.0355$
		main effect of treatment	$F (1, 69) = 4.519$	$p = 0.0371$
		main effect of age	$F (1, 69) = 2.462$	$p = 0.1212$
	Bonferroni's multiple comparisons test	Young adult control vs. Middle-aged control	$t (69.00) = 0.01298$	$p > 0.9999$
		Young adult control vs. Young adult with CoQ_{10}	$t (69.00) = 0.4219$	$p > 0.9999$
		Young adult control vs. Middle-aged with CoQ_{10}	$t (69.00) = 2.671$	$p = 0.0565$
		Middle-aged control vs. Young adult with CoQ_{10}	$t (69.00) = 0.3855$	$p > 0.9999$
		Middle-aged control vs. Middle-aged with CoQ_{10}	$t (69.00) = 2.538$	$p = 0.0804$
		Young adult with CoQ_{10} vs. Middle-aged with CoQ_{10}	$t (69.00) = 3.049$	$p = 0.0195$
Middle-aged mice data shown in Fig. 6a	paired *t* - test	the % change in the fEPSP amplitude		
		Control (averaged fEPSP amplitude, between -2 to 0 min and 58 to 60 min)	$t (10) = 1.412$	$p = 0.1883$
		CoQ_{10} (averaged fEPSP amplitude, between -2 to 0 min and 58 to 60 min)	$t (10) = 6.139$	$p = 0.0001$
		CoQ_{10} + APV (averaged fEPSP amplitude, between -2 to 0 min and 58 to 60 min)	$t (10) = 1.111$	$p = 0.2924$
Fig. 6b	one-way ANOVA	magnitude of LTP(%)	$F (2, 30) = 4.703$	$p = 0.0167$
	Bonferroni's multiple comparisons test	Control vs. CoQ_{10}	$t (30) = 2.694$	$p = 0.0343$
		Control vs. CoQ_{10} + APV	$t (30) = 0.07844$	$p > 0.9999$
		CoQ_{10} vs. CoQ_{10} + APV	$t (30) = 2.616$	$p = 0.0414$
Fig. 6c, Control	two-way repeated measures ANOVA	interaction (stimulus intensity x before/after)	$F (8, 80) = 5.145$	$p < 0.0001$
		main effect of stimulus intensity	$F (8, 80) = 102.2$	$p < 0.0001$
		main effect of before/after stumulation	$F (1, 10) = 0.6145$	$p = 0.4513$
	Bonferroni's multiple comparisons test	Before vs. After		
		Stimulus intensity: 10 μA	$t (80.00) = 1.300$	$p > 0.9999$
		Stimulus intensity: 20 μA	$t (80.00) = 1.929$	$p = 0.5153$
		Stimulus intensity: 30 μA	$t (80.00) = 0.4937$	$p > 0.9999$
		Stimulus intensity: 40 μA	$t (80.00) = 0.1597$	$p > 0.9999$
		Stimulus intensity: 50 μA	$t (80.00) = 2.073$	$p = 0.3722$
		Stimulus intensity: 60μA	$t (80.00) = 3.082$	$p = 0.0254$
		Stimulus intensity: 70 μA	$t (80.00) = 4.072$	$p = 0.0010$
		Stimulus intensity: 80 μA	$t (80.00) = 3.529$	$p = 0.0063$

		Stimulus intensity: 90 μA	t (80.00) = 3.050	p = 0.0279
Fig. 6c, CoQ_{10}	two-way repeated measures ANOVA	interaction (stimulus intensity x before/after)	F (8, 80) = 2.912	p = 0.0067
		main effect of stimulus intensity	F (8, 80) = 160.2	p < 0.0001
		main effect of before/after stumulation	F (1, 10) = 14.92	p = 0.0031
	Bonferroni's multiple comparisons test	Before vs. After		
		Stimulus intensity: 10 μA	t (80.00) = 3.743	p = 0.0031
		Stimulus intensity: 20 μA	t (80.00) = 4.607	p = 0.0001
		Stimulus intensity: 30 μA	t (80.00) = 5.590	p < 0.0001
		Stimulus intensity: 40 μA	t (80.00) = 6.202	p < 0.0001
		Stimulus intensity: 50 μA	t (80.00) = 6.969	p < 0.0001
		Stimulus intensity: 60μA	t (80.00) = 7.098	p < 0.0001
		Stimulus intensity: 70 μA	t (80.00) = 7.956	p < 0.0001
		Stimulus intensity: 80 μA	t (80.00) = 8.273	p < 0.0001
		Stimulus intensity: 90 μA	t (80.00) = 8.828	p < 0.0001
Fig. 6c, CoQ_{10} + APV	two-way repeated measures ANOVA	interaction (stimulus intensity x before/after)	F (8, 80) = 2.980	p = 0.0057
		main effect of stimulus intensity	F (8, 80) = 88.42	p < 0.0001
		main effect of before/after stumulation	F (1, 10) = 11.09	p = 0.0076
	Bonferroni's multiple comparisons test	Before vs. After		
		Stimulus intensity: 10 μA	t (80.00) = 1.632	p = 0.9599
		Stimulus intensity: 20 μA	t (80.00) = 4.278	p = 0.0005
		Stimulus intensity: 30 μA	t (80.00) = 4.647	p = 0.0001
		Stimulus intensity: 40 μA	t (80.00) = 4.534	p = 0.0002
		Stimulus intensity: 50 μA	t (80.00) = 2.965	p = 0.0359
		Stimulus intensity: 60μA	t (80.00) = 0.4009	p > 0.9999
		Stimulus intensity: 70 μA	t (80.00) = 2.516	p = 0.1248
		Stimulus intensity: 80 μA	t (80.00) = 1.058	p > 0.9999
		Stimulus intensity: 90 μA	t (80.00) = 0.3834	p > 0.9999

Supplementary Table 3. Summary of detailed data

Figure number/Data	Experiment	Value
Fig.1a left	Pole test, T-turn	
	Young adult control	1.50 ± 0.07 s
	Young adult CoQ_{10}	1.37 ± 0.06 s
	Middle-aged control	2.24 ± 0.13 s
	Middle-aged CoQ_{10}	1.66 ± 0.12 s
	Percentage decrease of T-turn values with CoQ_{10} supplementation to middle-aged mice	25.76%
Fig.1a right	Pole test, T-total	
	Young adult control	6.11 ± 0.27 s
	Young adult CoQ_{10}	5.77 ± 0.23 s
	Middle-aged control	6.23 ± 0.22 s
	Middle-aged CoQ_{10}	5.17 ± 0.22 s
	Percentage decrease of T-total values with CoQ_{10} supplementation to middle-aged mice	16.92%
Fig. 1c left	Pole test, T-turn	
	Middle-aged control	2.11 ± 0.11 s
	Middle-aged CoQ_{10}	1.50 ± 0.08 s
	Percentage decrease of T-turn values with CoQ_{10} supplementation for about one month to middle-aged mice	28.62%
Fig. 1c right	Pole test, T-total	
	Middle-aged control	6.56 ± 0.32 s
	Middle-aged CoQ_{10}	4.86 ± 0.30 s
	Percentage decrease of T-total values with CoQ_{10} supplementation for about one month to middle-aged mice	25.94%
Fig. 2	Brain mitochondrial respiration	
	Middle-aged control	43.38 ± 4.23 pmol/s・mg protein
	Middle-aged CoQ_{10}	65.17 ± 9.39 pmol/s・mg protein
Fig. 3a left (M1)	Percentage decrease of fEPSP amplitudes by aging	
	Stimulus intensity: 20 μA	40.51%
	Stimulus intensity: 30 μA	42.51%
	Stimulus intensity: 40 μA	36.24%
	Stimulus intensity: 50 μA	34.13%
	Stimulus intensity: 60μA	33.31%
	Stimulus intensity: 70 μA	32.12%
	Stimulus intensity: 80 μA	28.30%
	Average of percentage decrease of fEPSP amplitudes	35.30 ± 1.85%
Fig. 3b left (M1)	Percentage increase of fEPSP amplitudes by CoQ_{10} supplementation	
	Stimulus intensity: 20 μA	63.03%
	Stimulus intensity: 30 μA	63.48%
	Stimulus intensity: 40 μA	58.08%
	Stimulus intensity: 50 μA	64.23%
	Stimulus intensity: 60μA	64.63%
	Stimulus intensity: 70 μA	58.59%
	Stimulus intensity: 80 μA	58.55%
	Average of percentage increase of fEPSP amplitudes	61.51 ± 1.12%
Fig.5b	fEPSP amplitude (%)	
	without stim	102.34 ± 3.23%
	with stim	122.39 ± 7.15%
Fig.5d	magnitude of LTP (%)	
	Young adult control	108.10 ± 1.98%
	Middle-aged control	108.03 ± 1.35%
	Young adult CoQ_{10}	105.87 ± 1.84%

	Middle-aged CoQ_{10}	122.39 ± 7.15%
Fig. 6b	magnitude of LTP(%)	
	Control	102.70 ± 1.91%
	CoQ_{10}	110.88 ± 1.77%
	CoQ_{10} + APV	102.94 ± 2.65%
Fig. 6c, CoQ_{10}	Percentage of fEPSP amplitude (After) relative to fEPSP amplitude (Before)	
	Stimulus intensity: 10 μA	128.49%
	Stimulus intensity: 20 μA	116.97%
	Stimulus intensity: 30 μA	115.06%
	Stimulus intensity: 40 μA	113.89%
	Stimulus intensity: 50 μA	114.01%
	Stimulus intensity: 60μA	113.00%
	Stimulus intensity: 70 μA	113.90%
	Stimulus intensity: 80 μA	113.77%
	Stimulus intensity: 90 μA	114.42%
	Average of percentage of fEPSP amplitude (After) relative to fEPSP amplitude (Before)	115.95 ± 1.61%
